# Diet–lifestyle oxidative balance in relation to cardiometabolic multimorbidity: findings from the national health and nutrition examination survey

**DOI:** 10.3389/ebm.2025.10824

**Published:** 2025-12-18

**Authors:** Wenrui Shi, Yu Zhao, Jieun Park, Wan Chen

**Affiliations:** 1 Department of Cardiology, Shanghai Chest Hospital, Shanghai Jiao Tong University School of Medicine, Shanghai, China; 2 School of Medicine, Shanghai Jiao Tong University, Shanghai, China; 3 Department of Endocrinology, Yuhuan Second People’s Hospital, Yuhuan, Zhejiang, China

**Keywords:** oxidative stress, diet, oxidative balance score, cardiometabolic multimorbidity, NHANES

## Abstract

Oxidative stress is a critical factor in the development of cardiometabolic diseases. The Oxidative Balance Score (OBS), integrating dietary and lifestyle factors, has been proposed as a measure of the balance between pro-oxidants and antioxidants. This study aims to explore the relationship between OBS and prevalent cardiometabolic multimorbidity (CMM), and to evaluate whether adding OBS into clinical practice is associated with better CMM identification in the general population. A total of 26,191 participants were selected from the National Health and Nutrition Examination Survey. CMM was defined as having a history of two or more conditions: diabetes mellitus, stroke, or coronary heart disease. The prevalence of CMM was 2.95%. After adjusting for demographic, anthropometric, laboratory, and medical history data, each standard deviation increase in OBS was associated with a 26.1% reduction in the risk of prevalent CMM. Participants in the highest quartile of OBS had a 0.530-fold risk of prevalent CMM compared to those in the lowest quartile. Smooth curve fitting indicated a proportional reduction in CMM risk with increasing OBS. Sensitivity analysis confirmed significant associations between both dietary and lifestyle OBS with prevalent CMM. ROC analysis revealed that incorporating OBS into conventional cardiometabolic risk factors was associated with a slight improvement in CMM identification (AUC: 0.912 vs. 0.916, P = 0.001). Reclassification analysis further indicated the incremental value of OBS. This study revealed a negative, linear, and robust association between OBS and prevalent CMM in the general population. However, reverse causation cannot be ruled out. Future studies should use longitudinal or Mendelian randomization approaches to establish causality.

## Impact statement

Cardiometabolic multimorbidity (CMM) is an increasing health challenge with limited tools for early detection. This study shows that the Oxidative Balance Score (OBS), reflecting dietary and lifestyle factors, is strongly and inversely associated with CMM in a large national population. Importantly, incorporating OBS into conventional risk models was associated with improved CMM identification, highlighting its potential value for CMM identification. These findings advance the field by supporting OBS as a practical, quantifiable, and supplemental marker to assist the identification of CMM. The study highlights OBS as a potentially useful tool for guiding personalized lifestyle interventions, supporting prevention strategies, and helping to monitoring the burden of cardiometabolic diseases at the population level.

## Introduction

As global population aging and urbanization accelerate, the risk of individuals developing multiple chronic diseases—particularly cardiovascular disease (CVD) and its associated complications—continues to grow, leading to steadily increasing morbidity and mortality rates [1, 2]. Multimorbidity has emerged as a significant public health challenge due to its links to diminished quality of life, increased disability, and higher mortality rates [3–5]. Among the various forms of multimorbidity, cardiometabolic multimorbidity (CMM)—characterized by the presence of two or more conditions such as diabetes mellitus, stroke, and coronary heart disease (CHD)—is both the most prevalent and the most severe. Research indicated that individuals with CMM experience a reduction in life expectancy of 12–15 years by age 60 and face a 3.7–6.9 times higher risk of all-cause mortality compared to those without cardiometabolic conditions, with a significantly greater risk increase than those with only one such disease [3]. Given this serious situation, there is an urgent need to explore and expand the risk factor profile for CMM to facilitate its early detection and intervention.

Oxidative stress plays a pivotal role in endothelial dysfunction, atherosclerosis, and other pathogenic mechanisms contributing to the development of atherosclerotic CVD and diabetes [6, 7]. While substantial evidence links individual antioxidant or pro-oxidant exposure to CVD [8–10], few observational studies have explored the relationship between overall oxidative balance status and CVD. This gap may be due to the complexity of measuring oxidative balance-related exposures and the potential biological interactions between multiple pro-oxidants and antioxidants. Research indicates that lifestyle and dietary patterns can influence the body’s oxidative stress state [11]. In this context, the Oxidative Balance Score (OBS)—a metric assessing lifestyle and dietary factors—can be used to calculate behavioral scores and gauge antioxidant exposure levels [12]. Evidence strongly suggests a negative correlation between OBS and conditions such as diabetes [13], hypertension [14], chronic kidney disease [11], and CHD [15]. However, the relationship between OBS and the risk of CMM remains unclear.

Therefore, this study aimed to assess the relationship between OBS and the prevalence of CMM, and to evaluate whether adding OBS into clinical practice is associated with better CMM identification in the general population.

## Materials and methods

### Study design and population

The datasets for this study were derived from the National Health and Nutrition Examination Survey (NHANES) website, covering the years 1999–2018. NHANES is an ongoing program conducted by the National Center for Health Statistics that consists of a series of independent, nationally representative surveys. Using a cross-sectional design, NHANES has been conducted biennially in the United States for over 20 years. It employs a multistage, stratified, and clustered probability sampling strategy to ensure representativeness of the data. Data from different survey cycles are designed to be combinable for integrated analysis. Comprehensive details about NHANES, including its recruitment strategies, population coverage, and methodological design, are accessible on the Centers for Disease Control and Prevention (CDC) website.^1^

**FIGURE 1:**
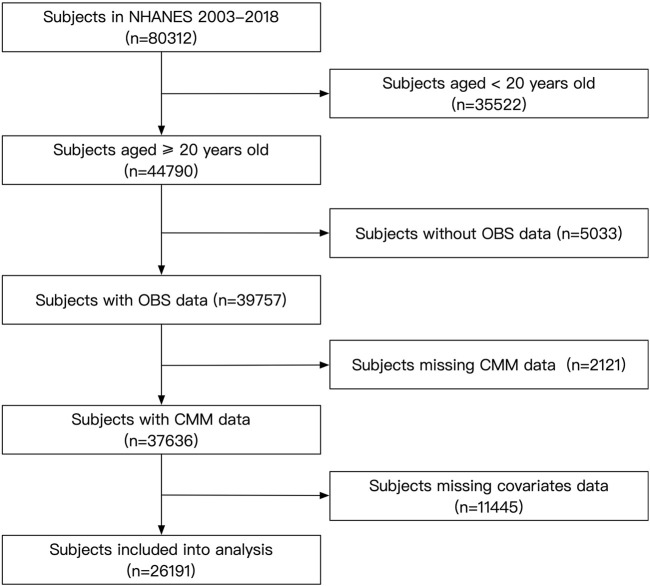
Flow chart of the subject’s enrollment.

For this analysis, we included subjects who participated in NHANES between 2003 and 2018 (N = 80,312). Exclusion criteria included individuals under 20 years of age and those with missing data on OBS, CMM, or other covariates. After applying these criteria, a total of 26,191 participants were included in the study (Figure 1). The NHANES protocol was approved by the NCHS Institutional Ethics Review Board, and as our study utilized de-identified data, no additional ethical review was required. All data used in this study are publicly available through the official NHANES website.

### Outcome ascertainment

CMM was defined as the presence of at least two of the following conditions: CHD, stroke, and diabetes [16, 17]. CHD was identified by a “yes” response to any of the questions: “Ever told you had coronary heart disease,” “Ever told you had angina,” or “Ever told you had a heart attack.” Stroke was determined by a “yes” response to the question “Ever told you had a stroke.” Receiving anti-diabetic therapy was indicated by a “yes” response to either “Take diabetic pills to lower blood sugar” or “Taking insulin now.” Diagnosed diabetes was defined as a “yes” response to “Doctor told you have diabetes.” Diabetes was classified based on fasting plasma glucose ≥7 mmol/L, receiving anti-diabetic therapy, or a diagnosis of diabetes [18].

### Exposure definition

The OBS was derived from 20 components, including 16 dietary and 4 lifestyle factors. Each factor was assigned a score between 0 and 2 based on predefined cut-offs. The classification of components as pro-oxidants (total fat, iron, alcohol, BMI, cotinine) or antioxidants (dietary fiber, carotene, riboflavin, niacin, vitamin B6, total folate, vitamin B12, vitamin C, vitamin E, calcium, magnesium, zinc, copper, selenium, and physical activity) was based on extensive prior literature. For pro-oxidants, higher values were associated with lower scores, while for antioxidants, higher values corresponded to higher scores. A higher OBS indicates a greater balance toward antioxidant behaviors, whereas a lower score reflects a pro-oxidant balance [19]. Detailed information about OBS score distribution and cut-off values was presented in Supplementary Table S1.

BMI, physical activity, smoking, and alcohol consumption were included as indicators in the Lifestyle OBS. BMI was calculated by dividing weight (kg) by height squared (m^2^). Physical activity data were collected using the NHANES Physical Activity Questionnaire, administered in participants’ homes by trained interviewers through the Computer-Assisted Personal Interview system. The questionnaire captured both work-related activities (vigorous and moderate intensity) and leisure-time physical activities (such as walking, cycling, and other moderate to vigorous activities). Physical activity levels were calculated based on established methodologies, using the product of activity frequency per week, activity duration, and the Metabolic Equivalent score [20]. Cotinine, the primary metabolite of nicotine, was used as a marker for active smoking and exposure to environmental tobacco smoke or passive smoking, due to its longer half-life in the bloodstream compared to nicotine [21]. Plasma cotinine levels have also been widely utilized in quantitative exposure assessment studies. Alcohol consumption was defined as the average number of alcoholic drinks consumed per day over the past 12 months on days when alcohol was consumed, encompassing all types of alcoholic beverages.

To provide a detailed analysis of dietary intake in the US population, trained dietary interviewers conducted face-to-face 24-hour dietary recall interviews with participants using data from NHANES. The interviews were conducted in private rooms at the NHANES Mobile Examination Center (MEC). Each dietary interview room at the MEC was equipped with a standardized set of measurement guides to help respondents accurately report the quantity of food consumed. These guides, explicitly developed for the NHANES setting, were designed to facilitate accurate dietary assessment for the non-institutionalized US civilian population. The National Center for Health Statistics (NCHS) oversaw sample design and data collection, while the United States Department of Agriculture Food Survey Research Group provided expertise on dietary survey methodology, data processing, and review.

### Covariates

Answering “Yes” to the question “Now taking prescribed medicine for hypertension” was determined as anti-hypertensive therapy; A mean systolic blood pressure (SBP) ≥ 140 mmHg, and/or a mean diastolic blood pressure (DBP) ≥ 90 mmHg, and/or anti-hypertensive therapy were indicated as hypertension [22]. Estimated glomerular filtration rate (eGFR) was calculated according to the Chronic Kidney Disease Epidemiology Collaboration (CKD-EPI) equation [23].

Laboratory tests were performed in CDC-certified laboratories. FPG was measured using the oxygen rate method on the Beckman DxC800 modular chemistry analyzer. Scr was assessed using the Jaffe rate method on the DxC800 modular chemistry platform. Blood lipid levels were quantified via enzymatic assays conducted on the Roche Modular P and Roche Cobas 6000 chemistry analyzers.

### Statistical analysis

This study employed statistical weighting to account for the complex design of the NHANES survey. Categorical variables were summarized as frequencies with 95% confidence intervals (CIs), while continuous variables were presented as means with 95% CIs. Group comparisons were conducted using Chi-square tests for categorical variables and t-tests for continuous variables. The analysis was divided into two main components. First, the association between OBS and the risk of prevalent CMM was investigated using multivariate logistic regression, with results expressed as odds ratios (ORs) and 95% CIs. OBS was analyzed both as a continuous variable, with effects reported per standard deviation (SD) change, and as a categorical variable divided into quartiles, with a P-for-trend analysis to examine whether ORs decreased significantly from quartile 1 to quartile 4. A generalized additive model with a spline smooth-fitting function was applied to explore the linearity of the association across the full OBS range. Sensitivity analyses were conducted to assess the associations of dietary and lifestyle OBS with CMM prevalence, and subgroup analyses tested the robustness of the logistic regression results across conventional subpopulations. Another sensitive analysis was used to evaluate the impact of including hypertension and obesity in the definition of CMM. This analysis aimed to assess the robustness of the observed associations between the OBS and CMM under an extended definition of CMM. Second, receiver operating characteristic (ROC) analysis and reclassification analysis, including the continuous net reclassification index (NRI) and integrated discrimination index (IDI), were performed to assess the association between OBS and the identification of prevalent CMM. To evaluate the clinical utility of OBS, decision curve analysis (DCA) was performed. DCA was used to assess the net benefit of the predictive models with and without the inclusion of OBS across a range of clinically relevant risk thresholds (0–0.4). Net benefit was calculated by considering the trade-off between true positives and false positives, with threshold values set at clinically relevant levels (5%–10% risk). The analysis was conducted to assess the incremental value of adding OBS to the clinical risk factor model. All statistical analyses were conducted using Stata (version 15.0), R (The R Foundation), and EmpowerStats (X&Y Solutions, Inc., Boston, MA, USA), with statistical significance defined as a two-tailed P-value less than 0.05.

## Results

### Characteristics of subjects

Characteristic data were summarized in Table 1. 772 of the 26,191 subjects were diagnosed with CMM. The overall mean age was 44.60 years, with significant differences across subgroups (P = 0.016). The proportion of males was 51.53%, with no significant variation between groups (P = 0.549). Racial distribution showed significant variation across OBS quartile groups (P < 0.001), with the proportion of non-Hispanic whites continuously increasing and that of non-Hispanic blacks steadily decreasing from quartile 1 to quartile 4. PIR also continuously rose from 2.70 in quartile 1 to 3.41 in quartile 4 (P < 0.001). For anthropometric parameters, height gradually increased, whereas weight, BMI, WC, SBP, and DBP consistently decreased from the lowest to the highest quartile. Regarding laboratory data, FPG, glycohemoglobin, LDL-C, and Scr decreased while HDL-C increased from the bottom quartile to the top quartile. The differences in TC, triglycerides, and eGFR among the quartile groups were significant but did not exhibit a clear trend. Regarding the medical history data, the percentages of individuals receiving anti-hypertensive therapy, anti-diabetic therapy, and those with diagnosed diabetes gradually decreased from quartile 1 to quartile 4, along with the prevalence rates of hypertension and diabetes. Meanwhile, the rates of diagnosed CHD and stroke followed the same pattern. Finally, the estimated prevalence of CMM decreased from 3.43% in quartile 1–1.21% in quartile 4 (P < 0.001).

**TABLE 1:** Characteristic profile of the enrolled subjects classified by OBS quartiles.

Variables	Total (26,191)	OBS quartile 1 (n = 6644)	OBS quartile 2 (n = 6593)	OBS quartile 3 (n = 7,005)	OBS quartile 4 (n = 5,949)	P value^a^
Age (years)	44.60 (44.10–45.09)	44.07 (43.42–44.72)	44.99 (44.32–45.67)	44.95 (44.33–45.57)	44.27 (43.56–44.98)	0.016
Male (%)	51.53 (50.88–52.17)	52.51 (51.03–53.99)	50.87 (49.45–52.29)	51.47 (49.98–52.96)	51.36 (49.73–52.99)	0.549
Race (%)						<0.001
Mexican American	7.69 (6.62–8.91)	7.16 (5.96–8.59)	7.97 (6.79–9.35)	7.62 (6.46–8.96)	7.96 (6.78–9.32)	
Other hispanic	4.73 (4.14–5.41)	4.98 (4.07–6.07)	4.90 (4.22–5.67)	4.43 (3.76–5.20)	4.69 (3.97–5.53)	
Non-hispanic white	71.15 (68.86–73.34)	66.16 (63.06–69.13)	70.61 (67.95–73.13)	72.78 (70.41–75.03)	74.31 (71.94–76.55)	
Non-hispanic black	9.67 (8.55–10.92)	15.31 (13.44–17.38)	10.20 (8.91–11.65)	8.07 (7.09–9.17)	5.92 (5.13–6.84)	
Others	6.76 (6.14–7.42)	6.39 (5.59–7.29)	6.32 (5.50–7.26)	7.10 (6.28–8.03)	7.12 (6.21–8.14)	
PIR	3.12 (3.05–3.18)	2.70 (2.63–2.77)	3.07 (3.00–3.14)	3.23 (3.16–3.30)	3.41 (3.32–3.49)	<0.001
Height (cm)	169.69 (169.51–169.86)	168.75 (168.45–169.05)	169.14 (168.83–169.45)	170.01 (169.69–170.33)	170.69 (170.34–171.04)	<0.001
Weight (kg)	82.17 (81.72–82.61)	84.30 (83.56–85.04)	82.94 (82.30–83.57)	82.39 (81.75–83.04)	79.24 (78.44–80.04)	<0.001
BMI (kg/m^2^)	28.45 (28.29–28.61)	29.52 (29.26–29.79)	28.91 (28.70–29.12)	28.41 (28.20–28.61)	27.09 (26.84–27.34)	<0.001
WC (cm)	97.76 (97.35–98.18)	100.31 (99.67–100.94)	98.81 (98.27–99.34)	97.78 (97.22–98.33)	94.45 (93.76–95.13)	<0.001
SBP (mmHg)	121.02 (120.66–121.37)	122.40 (121.75–123.04)	121.85 (121.32–122.39)	120.97 (120.42–121.51)	119.02 (118.49–119.54)	<0.001
DBP (mmHg)	70.98 (70.63–71.33)	71.32 (70.82–71.81)	70.94 (70.45–71.43)	71.23 (70.79–71.67)	70.45 (70.01–70.89)	0.003
FPG (mmol/L)	5.36 (5.34–5.39)	5.45 (5.40–5.50)	5.40 (5.36–5.45)	5.37 (5.32–5.43)	5.23 (5.19–5.28)	<0.001
Glycohemoglobin (%)	5.51 (5.50–5.53)	5.57 (5.54–5.60)	5.54 (5.51–5.56)	5.52 (5.49–5.54)	5.44 (5.41–5.47)	<0.001
TC (mmol/L)	5.02 (5.00–5.05)	5.04 (4.99–5.08)	5.04 (5.00–5.08)	5.04 (5.00–5.08)	4.98 (4.94–5.01)	0.010
Triglycerides (mmol/L)	1.66 (1.64–1.69)	1.69 (1.65–1.73)	1.68 (1.63–1.72)	1.71 (1.65–1.77)	1.57 (1.52–1.62)	0.001
LDL-C (mmol/L)	3.30 (3.28–3.32)	3.37 (3.33–3.42)	3.33 (3.30–3.36)	3.29 (3.26–3.32)	3.21 (3.18–3.24)	<0.001
HDL-C (mmol/L)	1.39 (1.38–1.40)	1.33 (1.31–1.34)	1.38 (1.36–1.39)	1.41 (1.39–1.42)	1.45 (1.43–1.47)	<0.001
Scr (μmol/L)	78.16 (77.72–78.60)	80.35 (79.40–81.29)	78.27 (77.60–78.94)	77.83 (77.17–78.48)	76.47 (75.83–77.11)	<0.001
eGFR (ml/min/1.73 m^2^)	96.04 (95.41–96.68)	96.06 (95.10–97.03)	95.60 (94.70–96.50)	95.71 (94.96–96.46)	96.85 (95.98–97.71)	0.028
Anti-hypertension therapy (%)	22.24 (21.36–23.15)	26.19 (24.73–27.71)	23.18 (21.54–24.90)	22.39 (20.96–23.88)	17.63 (16.36–18.97)	<0.001
Anti-diabetic therapy (%)	5.36 (5.01–5.72)	6.35 (5.59–7.21)	5.53 (4.85–6.30)	5.44 (4.81–6.15)	4.19 (3.58–4.90)	<0.001
Diagnosed diabetes (%)	6.91 (6.50–7.35)	8.43 (7.63–9.31)	7.19 (6.40–8.07)	6.85 (6.10–7.68)	5.36 (4.67–6.14)	<0.001
Hypertension (%)	29.16 (28.16–30.17)	33.04 (31.19–34.94)	30.74 (29.22–32.30)	29.31 (27.68–30.99)	23.94 (22.53–25.41)	<0.001
Diabetes (%)	10.78 (10.31–11.27)	12.55 (11.50–13.67)	11.79 (10.85–12.80)	10.56 (9.69–11.49)	8.46 (7.63–9.35)	<0.001
CHD (%)	4.53 (4.18–4.90)	6.62 (5.88–7.45)	4.64 (4.04–5.32)	3.96 (3.45–4.56)	3.18 (2.63–3.84)	<0.001
Stroke (%)	1.78 (1.59–2.00)	2.62 (2.18–3.14)	1.79 (1.45–2.21)	1.76 (1.41–2.19)	1.05 (0.78–1.41)	<0.001
CMM (%)	2.09 (1.88–2.33)	3.43 (2.94–3.99)	2.30 (1.87–2.83)	1.63 (1.38–1.93)	1.21 (0.92–1.59)	<0.001

Data were displayed as mean (95% confidence intervals) or numbers (95% confidence intervals) according to their data type.

^a^ Comparison of categorical variables was performed by Chi-square test. ANOVA was used for the comparison of continuous variables.

Abbreviations: OBS, oxidative balance score; PIR, poverty-to-income ratio; BMI, body mass index; WC, waist circumference; SBP, systolic blood pressure; DBP, diastolic blood pressure; FPG, fasting plasma glucose; TC, total cholesterol; LDL-C, low density lipoprotein cholesterol; HDL-C, high density lipoprotein cholesterol; Scr, serum cholesterol; eGFR, estimated glomerular filtration rate; CHD, coronary heart disease; CMM, cardiometabolic multimorbidity.

### Linear association between OBS and the prevalent CMM

**FIGURE 2:**
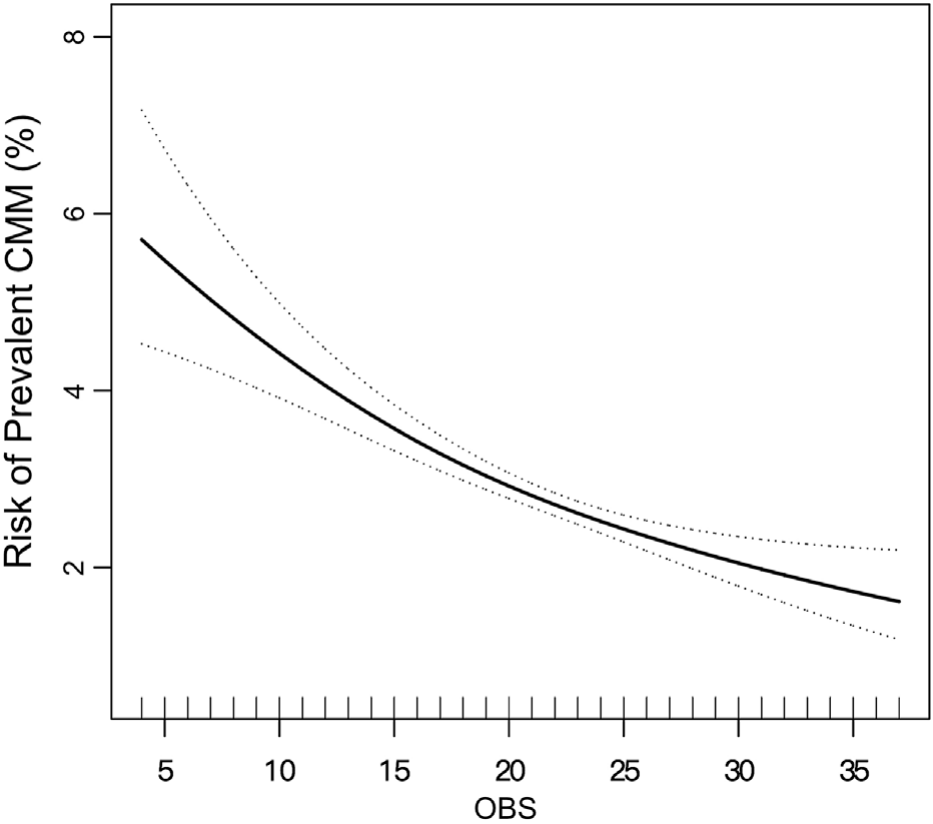
Smooth curve fitting illustrating the linear relationship between OBS and prevalent CMM. The model was adjusted for age, sex, race, PIR, BMI, WC, SBP, FPG, TC, HDL-C, eGFR, anti-hypertensive therapy, and anti-diabetic therapy, consistent with Model 2 in Table 2. The solid line represents the estimated risk of prevalent CMM, while the dotted lines indicate the pointwise 95% confidence intervals. The association remained linear across the entire range of OBS.

The results of the logistic regression analysis were presented in Table 2. Analyzing OBS as a continuous variable revealed that each SD increase was associated with a 35.1% reduction in CMM risk. After adjusting for age, sex, race, and PIR status, the risk reduction decreased to 31.5% per SD increase. Further adjustments for BMI, WC, mSBP, FPG, TC, HDL-C, eGFR, anti-hypertensive therapy, and anti-diabetic therapy reduced the risk reduction to 26.1%. When OBS was categorized into quartiles, the highest quartile showed a 0.530-fold risk of prevalent CMM compared to the lowest quartile in Model 2, with a clear trend of decreasing risk from quartile 1 to quartile 4 (P for trend <0.001). To validate this observed trend in the logistic regression analysis, a smooth curve fitting analysis was performed (Figure 2), which demonstrated a linear decrease in CMM risk across the entire range of OBS values.

**TABLE 2:** Association between OBS and the prevalent CMM.

Variables	Odds ratio (95% CI)					
	Crude	P value	Model 1	P value	Model 2	P value
OBS (per SD increase)	0.649 (0.587–0.718)	<0.001	0.685 (0.612–0.767)	<0.001	0.739 (0.654–0.835)	<0.001
Quartiles of OBS						
Quartile 1	Reference		Reference		Reference	
Quartile 2	0.663 (0.520–0.846)	0.001	0.687 (0.533–0.887)	0.004	0.741 (0.565–0.973)	0.031
Quartile 3	0.468 (0.370–0.592)	<0.001	0.509 (0.399–0.649)	<0.001	0.544 (0.418–0.707)	0.001
Quartile 4	0.345 (0.251–0.474)	<0.001	0.417 (0.298–0.582)	<0.001	0.530 (0.372–0.754)	<0.001
P for trend		<0.001		<0.001		<0.001

Crude: no adjustment.

Model 1: adjusted for demographic covariates (age sex race PIR).

Model 2: further adjusted for anthropometric, laboratory, and medical history data (BMI, WC, SBP, FPG, TC, LDL-C, HDL-C, eGFR, anti-hypertensive therapy, anti-diabetic therapy).

Abbreviations: OBS, oxidative balance score; CMM, cardiometabolic multimorbidity; OR, odds ratio; CI, confidence interval; SD, standard deviation; PIR, poverty-to-income ratio; BMI, body mass index; WC, waist circumference; SBP, systolic blood pressure; FPG, fasting plasma glucose; TC, total cholesterol; LDL-C, low density lipoprotein cholesterol; eGFR, estimated glomerular filtration rate.

### Sensitivity analysis for the association between OBS and the prevalent CMM

We further explored the associations between dietary OBS, lifestyle OBS, and prevalent CMM (Table 3), using the same adjustment strategy as in Table 2. Consistent with the findings for OBS, each SD increase in dietary OBS was associated with a 24.5% reduction in the risk of prevalent CMM. Additionally, individuals in the highest quartile of dietary OBS had a 0.513-fold risk of prevalent CMM compared to those in the lowest quartile, with a clear linear trend of decreasing risk from quartile 1 to quartile 4 (P for trend <0.001). In contrast, while higher lifestyle OBS was also linked to a reduced risk of CMM, the risk reduction did not follow a linear pattern across the quartiles.

**TABLE 3:** Association between dietary and lifestyle OBS and the prevalent CMM.

Variables	Odds ratio (95% CI)					
	Crude	P value	Model 1	P value	Model 2	P value
Dietary OBS (per SD increase)	0.672 (0.609–0.741)	<0.001	0.730 (0.656–0.813)	<0.001	0.755 (0.670–0.850)	<0.001
Quartiles of dietary OBS						
Quartile 1	Reference		Reference		Reference	
Quartile 2	0.677 (0.536–0.855)	0.001	0.722 (0.561–0.929)	0.012	0.732 (0.556–0.963)	0.026
Quartile 3	0.481 (0.368–0.630)	<0.001	0.556 (0.423–0.731)	<0.001	0.567 (0.424–0.758)	0.001
Quartile 4	0.339 (0.238–0.483)	<0.001	0.447 (0.308–0.648)	<0.001	0.513 (0.351–0.749)	<0.001
P for trend		<0.001		<0.001		<0.001
Lifestyle OBS (per SD increase)	0.782 (0.716–0.854)	<0.001	0.696 (0.633–0.765)	<0.001	0.840 (0.744–0.949)	0.005
Quartiles of lifestyle OBS						
Quartile 1	Reference		Reference		Reference	
Quartile 2	0.711 (0.546–0.925)	0.012	0.588 (0.449–0.769)	<0.001	0.658 (0.500–0.866)	0.003
Quartile 3	0.836 (0.649–1.076)	0.162	0.672 (0.518–0.872)	0.003	0.844 (0.643–1.107)	0.219
Quartile 4	0.409 (0.299–0.559)	<0.001	0.315 (0.229–0.432)	<0.001	0.635 (0.442–0.912)	0.014
P for trend		<0.001		<0.001		0.048

Crude: no adjustment.

Model 1: adjusted for demographic covariates (age sex race PIR).

Model 2: further adjusted for anthropometric, laboratory, and medical history data (BMI, WC, SBP, FPG, TC, LDL-C, HDL-C, eGFR, anti-hypertensive therapy, anti-diabetic therapy).

Abbreviations: OBS, oxidative balance score; CMM, cardiometabolic multimorbidity; OR, odds ratio; CI, confidence interval; SD, standard deviation; PIR, poverty-to-income ratio; BMI, body mass index; WC: waist circumference; SBP, systolic blood pressure; FPG, fasting plasma glucose; TC, total cholesterol; LDL-C, low density lipoprotein cholesterol; eGFR, estimated glomerular filtration rate.

**FIGURE 3:**
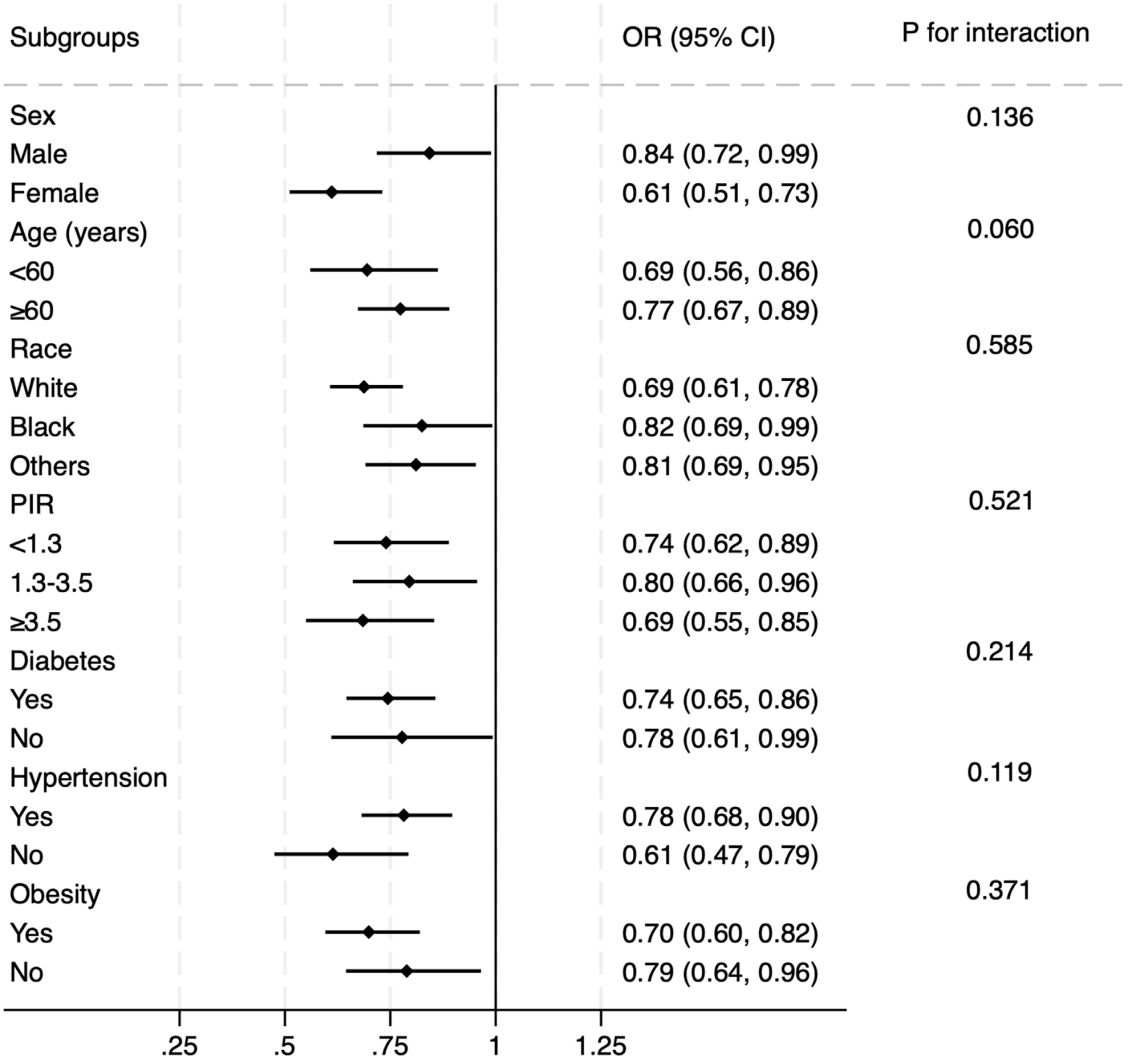
Subgroup analysis of the correlation between OBS and prevalent CMM. The multivariate logistic model was adjusted for all variables included in Model 2 of Table 2, except the variable used to define each subgroup. The association remained consistent across subgroups defined by sex, age, race, diabetes, hypertension, and obesity.

Subgroup analysis was further performed to evaluate the effectiveness of the results observed in the overall population across various conventional subpopulations (Figure 3). Logistic regression models were adjusted for all covariates included in Model 2, excluding those used to define the subgroups. The findings confirmed that the association between OBS and prevalent CMM remained robust across subgroups defined by sex, age, race, PIR, diabetes, hypertension, and obesity (all P values for interaction >0.05). In Supplementary Table S2, we added hypertension and obesity to the definition of CMM. Similar results were observed under the same adjusting strategy. In model 2, each SD increase in OBS was associated with a 12.3% decrease in the risk of prevalent CMM.

### Association of OBS with the identification of prevalent CMM

ROC and reclassification analyses were conducted to assess the association between OBS and the identification of prevalent CMM (Table 4). In the ROC analysis, the AUC for OBS alone was 0.622 (95% CI: 0.616–0.628). Adding OBS to the clinical risk factors (covariates from Model 2 in Table 2) was associated with a slight increase in the AUC from 0.912 to 0.916 (P = 0.001). Reclassification analysis further indicated this association, with a continuous NRI of 0.243 (95% CI: 0.172–0.314, P < 0.001) and an IDI of 0.007 (95% CI: 0.004–0.010, P < 0.001), suggesting that OBS may be associated with improved identification of prevalent CMM. DCA also showed a significant improvement when OBS was added to clinical risk factors (Supplementary Figure S1). The results showed that adding OBS to the clinical risk factors model was associated with an increase in net benefit across a range of clinically relevant risk thresholds (0–0.4).

**TABLE 4:** Assessment of the value of OBS for detecting prevalent CMM.

Model	AUC (95% CI)	P value	P for comparison	NRI (continuous)	P value	IDI	P value
OBS	0.622 (0.616–0.628)	<0.001	-	-	-	-	-
Clinical risk factors^a^	0.912 (0.908–0.915)	<0.001	-	-	-	-	-
Clinical risk factors + OBS	0.916 (0.913–0.920)	<0.001	0.001	0.243 (0.172–0.314)	<0.001	0.007 (0.004–0.010)	<0.001

^a^ Clinical risk factors: age, sex, race, PIR, BMI, WC, SBP, FPG, TC, HDL-C, eGFR, anti-hypertensive therapy, and anti-diabetic therapy.

Abbreviations: OBS, oxidative balance score; CMM, cardiometabolic multimorbidity; AUC, area under the curve; NRI, net reclassification index; IDI, integrated discrimination index; PIR, poverty-to-income ratio; BMI, body mass index; WC, waist circumference; SBP, systolic blood pressure; FPG, fasting plasma glucose; TC, total cholesterol; LDL-C, low density lipoprotein cholesterol; eGFR, estimated glomerular filtration rate.

## Discussion

In the current analysis, our findings revealed a significant association between OBS and prevalent CMM in the general population. This relationship was negatively linear across the entire range of OBS, indicating that the risk of prevalent CMM increased proportionally with higher OBS values. The association remained consistent after adjusting for common cardiovascular risk factors, demonstrating its robustness across various subpopulations. Furthermore, the addition of OBS to conventional cardiovascular risk factors achieved significance improvement in ROC and reclassification analysis, suggesting that applying OBS into clinical practice may be associated with improved identification of prevalent CMM in the general population.

### The linear association between OBS and the risk of prevalent CMM

The findings from this analysis supported the hypothesis of a significant association between OBS and prevalent CMM, highlighting the potential utility of OBS in detecting CMM in the general population. The first part of the analysis focused on this association, using multivariate logistic regression adjusted for demographic, laboratory, anthropometric, and medical history variables. The results demonstrated a significant and independent relationship between OBS and prevalent CMM, unaffected by conventional cardiovascular risk factors. Assuming a linear association, a smooth curve-fitting analysis was performed, confirming that the relationship between OBS and prevalent CMM was linearly negative across the entire OBS range. This indicated that the risk of prevalent CMM increased proportionally with higher OBS values, without evidence of a threshold or saturation effect.

The analysis also explored the individual contributions of dietary and lifestyle OBS to prevalent CMM. Dietary OBS exhibited a significant and linear association with CMM, with a slightly higher effect size (OR) compared to total OBS. In contrast, lifestyle OBS, while significantly associated with CMM, did not show a linear relationship. This discrepancy may be due to the limited number of elements in the lifestyle OBS (physical activity, alcohol, BMI, cotinine), with scores ranging only from 0 to 8, which could potentially affect the linearity due to score distribution and sample size. Further research is needed to determine whether the association between lifestyle OBS and CMM follows a non-linear pattern.

Finally, a subgroup analysis was conducted to evaluate whether the findings were consistent across key cardiovascular subpopulations defined by age, gender, race, socioeconomic status, diabetes, hypertension, and obesity. The results showed that the main findings from the overall population were robust and applicable across these subpopulations, reinforcing the generalizability of the observed association between OBS and prevalent CMM.

In the current study, we defined CMM as the coexistence of two or more conditions: diabetes mellitus, stroke, or coronary heart disease. This definition was based on prior literature [16, 17], which identified these conditions as key components of CMM. While hypertension and obesity are also major cardiometabolic conditions, they were not included in the primary definition due to their potential impact on the event prevalence. Including hypertension and obesity would have led to a dramatic increase in CMM prevalence from 2.95% to 23.70%, violating a key assumption of logistic regression that event prevalence should remain below 15%. This change could result in inflated odds ratios and distorted estimates. Nevertheless, we still conducted a sensitivity analysis that included hypertension and obesity in the CMM definition. The results of this analysis, presented in Supplementary Table S2, were consistent with our primary findings, indicating that the associations between OBS and CMM remained robust. These findings suggest that our conclusions are not influenced by the exclusion of hypertension and obesity.

### Association of OBS with the identification of prevalent CMM

In the second part of our statistical analysis, we shifted focus to evaluating the association between OBS and improved identification of prevalent CMM in the general population. To this end, both ROC, reclassification, and DCA analyses were employed to assess the supplemental value of OBS from a different perspective. While the AUC for OBS alone in identifying prevalent CMM was modest, incorporating OBS into conventional cardiovascular risk factors significantly improved the model’s ability to identify prevalent CMM. The increase in AUC is also limited, suggesting that OBS functions primarily as an additional and supplemental tool, rather than a replacement, for traditional risk factors. By assessing dietary and lifestyle factors, OBS helps identify individuals who may not be captured by conventional cardiovascular risk factors alone. This highlights OBS’s potential to identify individuals who might otherwise be overlooked by traditional models, offering a more comprehensive approach to CMM, particularly in relation to dietary and lifestyle habits.

However, it is important to note that ROC analysis evaluates the overall performance of the combined model (cardiovascular risk factors + OBS) rather than isolating the specific contribution of OBS to improving detection. As such, ROC analysis may overestimate or underestimate the individual value of OBS, providing limited insight into whether adding OBS to conventional cardiovascular risk factors meaningfully refines the detection of prevalent CMM [24, 25]. To address this limitation, reclassification analysis—using metrics such as NRI and IDI—has been proposed as an alternative approach to assess the added value of novel markers [26–28].

In our study, introducing OBS into the cardiovascular risk factors model resulted in significant improvements in both continuous NRI and IDI. While these values (NRI: 0.243 and IDI: 0.007) represent modest changes, they suggest better refinement in identifying prevalent CMM and demonstrate that OBS contributes incremental value to the existing model. The NRI suggests that OBS facilitates more accurate reclassification of individuals into categories, potentially enabling more targeted interventions. Similarly, while the IDI value was small, it reflects a refinement in the model’s discriminatory power, which may have important practical implications for clinical decision-making. These results, supported by both ROC and reclassification analyses, implicate OBS’s potential as a supplementary marker capable of identifying CMM patients who might otherwise be overlooked by traditional models, offering a more comprehensive approach to CMM identification.

As highlighted in the literature, even small increases in AUC are often considered meaningful in clinical practice, as they can lead to better model discrimination between high and low-risk individuals. Baker et al. noted that even a slight AUC improvement could substantially impact clinical decision-making by enhancing the model’s ability to identify at-risk patients with greater accuracy [29]. To better assess the practical value of this slight increase in AUC, we further conducted DCA (Supplementary Figure S1). In our results, we observed that adding OBS to the clinical risk factor model improved the net benefit across a range of thresholds. This phenomenon demonstrated that OBS could still provide some valuable information to the model and function as a supplemental tool. In conclusion, the slight increase in AUC is clinically relevant, as supported by the DCA. Applying OBS in clinical practice could be associated with improved clinical decision-making. This finding underscores the potential value of OBS as a supplemental marker to identify prevalent CMM in clinical practice.

### Mechanisms underlying the association between OBS and CMM

Both dietary components of OBS play their role in the development and progression of cardiometabolic diseases. Vitamin C intake is significantly associated with a reduced risk of CHD and heart failure [30]. A high intake of vitamin E may lower the risk of CHD by preventing the formation of oxidized LDL-C, which can trigger endothelial cells to produce inflammatory markers, exert cytotoxic effects on these cells, and impair nitric oxide-mediated vasodilation [31]. Additionally, combining vitamin C with other antioxidants, such as vitamin E, leads to improved antioxidant effectiveness. Selenium, a vital component of glutathione peroxidase, plays a crucial role in protecting aerobic tissues from oxidative damage caused by free radicals during myocardial ischemia [32]. Magnesium deficiency, linked to the onset and progression of atherosclerotic injury, can trigger oxidative stress in the body. Magnesium intake enhances the vasodilatory effects of both endogenous and exogenous vasodilators and reduces cardiovascular risk by inhibiting platelet function [32]. Antioxidants also play a vital role in mitigating oxidative stress induced by reactive oxygen species, which are primarily triggered by uncontrolled high blood sugar levels in diabetes. Supplements such as vitamin C, vitamin E, selenium, and alpha-lipoic acid have demonstrated potential in reducing oxidative stress markers and enhancing antioxidant status in laboratory studies, animal models, and diabetic patients. Antioxidant supplementation has also been shown to improve endothelial function, insulin sensitivity, and glucose metabolism, contributing to better glycemic control and overall management of diabetes [7].

### Clinical implications

The primary clinical implication of this study is the detailed characterization of the association between OBS and prevalent CMM, which reinforces the link between oxidative balance and CMM. OBS, as a measure of oxidative stress balance, was found to have a linear association with the risk of prevalent CMM, suggesting that managing oxidative stress may be associated with a lower likelihood of CMM. Another key implication is that applying OBS in clinical practice could be associated with improved identification of prevalent CMM, particularly in primary care settings. CMM is one of the most prevalent and hazardous forms of multimorbidity, associated with reduced life expectancy and increased mortality, making it a significant cardiovascular risk factor, especially in older populations. However, CMM is often overlooked, particularly in primary care, where its identification is critical for cardiovascular prevention. This issue is more pronounced in rural areas of developing countries, where many individuals with cardiometabolic diseases remain undiagnosed, and essential treatment is lacking. The identification rate of CMM is notably low in these populations.

Our findings suggest that OBS could be associated with improved identification of prevalent CMM in the general population. Given its cost-effectiveness, integrating OBS into clinical practice could improve the identification of CMM in primary care settings. This, in turn, would enable general practitioners to deliver more personalized medical care for patients with CMM, addressing an unmet need in managing this high-risk condition.

### Limitations

Our study has some limitations. First, a major limitation of this study is the cross-sectional design, which prevents us from establishing causality. Individuals with existing cardiometabolic conditions could have altered their diet or lifestyle in response to their health status, potentially influencing their OBS. Therefore, while we observe an association between OBS and CMM, future studies utilizing longitudinal data or Mendelian randomization approaches are needed to better establish causal relationships. These methods would help to confirm whether OBS directly influences the risk of CMM or if the relationship is due to reverse causation. Second, as shown in Figure 1. 18,599 participants were excluded due to missing data related to key variables, including OBS, CMM, and other covariates. While we focused on complete cases to maintain the integrity of the analysis, the excluded participants may differ systematically from those included in the study, potentially leading to selection bias. For instance, those excluded due to missing OBS data, CMM data, or covariate data might represent different demographic or clinical groups, which could affect the generalizability of our results. This selection bias could lead to over- or underestimation of the association between the OBS and CMM, particularly in the underrepresented groups. Although multiple imputation is a standard method for handling missing data, it was not applied in this study due to the complexity of imputing OBS data, which involves a combination of dietary, lifestyle, and clinical factors that may not be easily modeled with the available data. Additionally, the assumptions required for multiple imputation, such as missing data being missing at random, were difficult to verify given the nature of the missing data in our dataset. Given these challenges, we chose to exclude participants with missing data to ensure the validity and robustness of our analysis. However, the potential impact of this exclusion on the overall findings remains an important consideration. Therefore, our findings still require future studies with more complete data collection to verify.

Third, the reliance on self-reported data in NHANES raises concerns about recall bias and subjectivity, potentially compromising data accuracy. Further studies using more reliable and objective definitions are required to validate our conclusions. Fourth, while we adjusted for a range of covariates, unmeasured confounders may still have influenced our results. Therefore, studies with more comprehensive data collection are needed to confirm our findings. Last, as NHANES was conducted exclusively in the United States, the generalizability of our results to other populations remains uncertain. Additional research involving diverse populations is essential to verify the applicability of our findings.

## Data Availability

The datasets presented in this study can be found in online repositories. The names of the repository/repositories and accession number(s) can be found below: The data used in the current study could be acquired from WC with a reasonable request. The data could also be downloaded from the NHANES official website (https://www.cdc.gov/nchs/nhanes/index.htm).

## Footnotes

1
https://www.cdc.gov/nchs/nhanes/index.htm
