# Supplementary material for: Diet–lifestyle oxidative balance in relation to cardiometabolic multimorbidity: findings from the national health and nutrition examination survey
Source: Exp Biol Med (Maywood). 2025 Dec 18;250:10824. doi: 10.3389/ebm.2025.10824 (PMC12756176; doi:10.3389/ebm.2025.10824)
Supplement: Supplementary file 1 [file Supplementaryfile1.docx]

Table S1: Components of the oxidative balance score.

| OBS components | Property | Male | | | Female | | |
| --- | --- | --- | --- | --- | --- | --- | --- |
|  |  | 0 | 1 | 2 | 0 | 1 | 2 |
| Dietary OBS components | | | | | | | |
| Dietary fiber (g/d) | A | <12.30 | 12.30-20.70 | ≥20.70 | <10.40 | 10.40-17.20 | ≥17.20 |
| Carotene (RE/d) | A | <36.65 | 36.65-124.35 | ≥124.35 | <34.42 | 34.42-141.99 | ≥141.99 |
| Riboflavin (mg/d) | A | <1.71 | 1.71-2.67 | ≥2.67 | <1.33 | 1.33-2.06 | ≥2.06 |
| Niacin (mg/d) | A | <21.69 | 21.69-33.26 | ≥33.26 | <15.43 | 15.43-23.59 | ≥23.59 |
| Vitamin B_6_ (mg/d) | A | <1.63 | 1.63-2.59 | ≥2.59 | <1.19 | 1.19-1.90 | ≥1.90 |
| Total folate (mcg/d) | A | <314.00 | 314.00-505.00 | ≥505.00 | <244.00 | 244.00-390.00 | ≥390.00 |
| Vitamin B_12_ (mcg/d) | A | <3.27 | 3.27-6.37 | ≥6.37 | <2.27 | 2.27-4.49 | ≥4.49 |
| Vitamin C (mg/d) | A | <31.10 | 31.10-99.00 | ≥99.00 | <30.10 | 30.10-89.94 | ≥89.94 |
| Vitamin E (ATE) (mg/d) | A | <5.59 | 5.59-9.69 | ≥9.69 | <4.64 | 4.64-7.96 | ≥7.96 |
| Calcium (mg/d) | A | <687.00 | 687.00-1164.00 | ≥1164.00 | <578.00 | 578.00-962.00 | ≥962.00 |
| Magnesium (mg/d) | A | <251.00 | 251.00-371.00 | ≥371.00 | <201.00 | 201.00-296.00 | ≥296.00 |
| Zinc (mg/d) | A | <9.30 | 9.30-14.91 | ≥14.91 | <6.88 | 6.88-10.74 | ≥10.74 |
| Copper (mg/d) | A | <1.02 | 1.02-1.53 | ≥1.53 | <0.83 | 0.83-1.25 | ≥1.25 |
| Selenium (mcg/d) | A | <96.85 | 96.85-148.20 | ≥148.20 | <70.60 | 70.60-108.20 | ≥108.20 |
| Total fat (g/d) | P | ≥107.80 | 67.95-107.80 | <67.95 | ≥80.86 | 51.16-80.86 | <51.16 |
| Iron (mg/d) | P | ≥18.86 | 12.16-18.86 | <12.16 | ≥14.36 | 9.26-14.36 | <9.26 |
| Lifestyle OBS components | | | | |  |  |  |
| Physical activity (MET-minute/week) | A | <960.00 | 960.00-4160.00 | ≥4160.00 | <607.40 | 607.40-2280.00 | ≥2280.00 |
| Alcohol (g/d) | P | ≥28 | 0-28 | None | ≥14 | 0-14 | None |
| Body mass index (kg/m^2^) | P | ≥29.80 | 25.40-29.80 | <25.40 | ≥30.93 | 24.85-30.93 | <24.85 |
| Cotinine (ng/mL) | P | ≥2.745 | 0.027-2.745 | <0.027 | ≥0.116 | 0.017-0.116 | <0.017 |

OBS: oxidative balance score; A: antioxidant; P: prooxidant; RE: retinol equivalent; ATE: alpha-tocopherol equivalent; MET: metabolic equivalent.

Table S2. Association between OBS and the prevalent CMM (extended definition).

| Variables | Odds Ratio (95% CI) | | | | | |
| --- | --- | --- | --- | --- | --- | --- |
|  | Crude | P value | Model 1 | P value | Model 2 | P value |
| OBS (Per SD increase) | 0.776 (0.743-0.711) | <0.001 | 0.772 (0.736-0.809) | <0.001 | 0.877 (0.825-0.934) | <0.001 |
| Quartiles of OBS |  |  |  |  |  |  |
| Quartile 1 | Reference |  | Reference |  | Reference |  |
| Quartile 2 | 0.799 (0.708-0.902) | <0.001 | 0.772 (0.676-0.881) | <0.001 | 0.780 (0.667-0.912) | 0.002 |
| Quartile 3 | 0.728 (0.648-0.817) | <0.001 | 0.714 (0.627-0.812) | <0.001 | 0.759 (0.647-0.891) | 0.001 |
| Quartile 4 | 0.513 (0.451-0.583) | <0.001 | 0.514 (0.446-0.592) | <0.001 | 0.723 (0.604-0.864) | <0.001 |
| P for trend |  | <0.001 |  | <0.001 |  | <0.001 |

Crude: no adjustment.

Model 1: adjusted for age, sex, race, and PIR.

Model 2: Model 1 + BMI, WC, SBP, FPG, TC, LDL-C, HDL-C, eGFR, anti-hypertensive therapy, anti-diabetic therapy.

CMM extended definition: the presence of at least two of the following conditions: coronary heart disease, stroke, diabetes, hypertension, and obesity.

Abbreviations: OBS: oxidative balance score; CMM: cardiometabolic multimorbidity; OR: odds ratio; CI: confidence interval; SD: standard deviation; PIR: poverty-to-income ratio; BMI: body mass index; WC: waist circumference; SBP: systolic blood pressure; FPG: fasting plasma glucose; TC: total cholesterol; LDL-C: low density lipoprotein cholesterol; eGFR: estimated glomerular filtration rate.

Figure S1. Comparison of net benefit with and without OBS using decision curve analysis.

This figure presents the net benefit of the clinical risk factor model for identifying prevalent CMM, with and without the inclusion of OBS, across a range of clinically relevant risk thresholds (0–0.4). The blue solid line represents the net benefit of the clinical risk factor model, while the red dotted line shows the net benefit of the model with OBS included. The gray line represents the net benefit of the "treat-all" strategy, while the black line indicates the "treat-none" strategy. The analysis suggests that adding OBS to clinical risk factors is associated with improvement of the model’s ability to identify prevalent CMM.
